# Can postmortem MRI be used to assess trajectories in gunshot victims?

**DOI:** 10.1007/s00414-015-1273-4

**Published:** 2015-10-30

**Authors:** M. Luijten, I. I. H. Haest, R. A. T. van Kan, W. van Lohuizen, J. Kroll, R. S. Schnerr, R. Hermsen, P. A. M. Hofman

**Affiliations:** Dutch Police, Region Limburg, PO Box 1230, 6201 BE Maastricht The Netherlands; Department of Radiology, Maastricht University Medical Center, PO. Box 5800, 6202 AZ Maastricht, The Netherlands; Netherlands Forensic Institute, PO Box 24044, 2490 AA The Hague, The Netherlands

**Keywords:** Projectile, MR imaging, Gunshot, Forensic radiology, Postmortem imaging, Safety

## Abstract

**Purpose:**

Multi-detector computed tomography (MDCT) has proven to be of value for the reconstruction of trajectories of projectiles and the assessment of the injuries in deceased gunshot victim. For the depiction of soft tissue injury, MRI is superior to MDCT and MRI may be of value to assess trajectories. In a clinical setting, there are guidelines for the application of MRI in patients with projectiles or projectile fragments and with precautions MRI is safe for these patients. However, this has not been studied for the postmortem application of MRI from a forensic point of view.

**Subjects and method:**

To assess the behaviour of projectiles, two ferromagnetic and one non-ferromagnetic projectile were exposed to the magnetic field of a 1.5- and 3-T MRI. Projectiles were placed in six phantoms with the characteristics of human muscle tissue, with and without a simulated trajectory in the gel. Before and after exposure to the magnetic field, the gelatine phantoms were imaged with MDCT to assess the position of the projectiles.

**Results:**

The ferromagnetic projectiles rotate to a position where their long axis is parallel to the *z*-axis of the magnetic field and five out of the six projectiles moved through, either through the simulated trajectory or through a new trajectory. This was observed in both the 1.5- and 3-T systems.

**Conclusion:**

Ferromagnetic projectiles can rotate and migrate in a gelatine phantom. It is very likely that these projectiles will also migrate in a human body in a MRI system. Therefore, from a forensic point of view, postmortem MR will make a reconstruction of the trajectories in the body and of the reconstruction of the incident as a whole less reliable.

## Introduction

Medical imaging techniques have shown to be a valuable tool for the postmortem examination of human remains, especially of crime victims. Forensic radiology provides information that may be difficult to assess otherwise and there is increasing evidence for the complementary and additional value of a forensic radiological examination [1, 2]. Multi-detector computed tomography (MDCT) is the most often used technique because it provides a depiction of body lesions and depicts metal objects very accurately. MDCT also allows for multi-planar and 3D reconstructions, which is necessary for the evaluation of complex lesions. More in particular, postmortem MDCT can be valuable for the assessment of trajectories of projectiles because radiology allows for the evaluation of the undisturbed anatomy, compared to the eviscerated organs during a forensic postmortem. This also makes it possible to test different hypothesis and reanalyse trajectories [3]. However for the assessment of soft tissue injury, MRI is superior to MDCT and the trajectories in soft tissue are better depicted in a MR examination of the body [2]. The presence of ferromagnetic objects in a body is a relative contraindication for a clinical MRI because the displacement of these objects under the influence of the static external magnetic field of a MRI scanner may harm the patient. In our experience, up to 60 % of victims have retained projectiles or projectile fragments and approximately 20 % of these projectiles are ferromagnetic. Guidelines have been proposed for the clinical application of MRI in the presence of ferromagnetic objects [4–6]. However, this has not been studied from a forensic point of view.

The purpose of our study is to analyse the possible movement of projectiles in a 1.5- and 3-T clinical MRI system using a gelatine phantom in order to propose a guideline for the application of MRI in the examination of deceased gunshot victims.

## Materials and methods

For the simulation of human tissue, a firm ballistic gelatine phantom was used. The gelatine phantom was prepared by dissolving 10 % gelatine in water of 45 °C [7, 8]. This mixture was cooled to 4 °C before use. Six gelatine phantoms of 1.7 L were prepared. The phantoms were used at 4 °C because at this temperature the density and viscosity of this gelatine is equal to the density and viscosity of muscle tissue [8]. Three different types of projectiles were used. From the database of the Netherlands Forensic Institute, a projectile was selected with a large amount of ferromagnetic steel. The second projectile had no ferromagnetic steel (negative control). The third projectile was completely made of ferromagnetic steel (see Table 1). The projectiles were placed in the gelatine phantom in two ways. With the first method, the projectile was suspended in the gelatine using a plastic wire while the gelatine phantom was still liquid. After 24 h the wire was removed without moving the projectile. With the second method, the projectile was pushed into the gelatine phantom in order to simulate a trajectory. All phantoms were initially imaged on a MDCT scanner (Somatom Definition Flash, Siemens Healthcare, Forchheim Germany) to assess the position of the embedded projectiles. The gelatine phantoms were placed in the middle of the table and moved in and out of the centre of the homogeneous magnetic field using the patient table. The maximum table speed was 0.2 m/s. After exposure to the magnetic field of the MRI scanner, the phantoms were rescanned with MDCT to detect and measure changes in the position of the projectiles. In between scanning sessions, the gelatine phantoms were stored at 4 °C and after the first and the longest scanning session the core temperature of the gelatine phantom was measured by introducing a thermometer in the gel. The position of the projectiles with regard to main axis (*z*-axis) of the static magnetic field of the MRI scanner was either perpendicular or parallel with the long axis of the projectiles. The clinically most prevailing magnetic field strengths were used (1.5 and 3 T). Only in the first experiment an imaging pulse sequence was employed. In order to increase the MRI signal a water phantom was scanned together with the gelatine phantom. For the other phantoms, only the effect of the B0 field was studied. In the first experiment, two gelatine phantoms with embedded projectile type A and a simulated trajectory (see Table 2) were placed in a 3-T MRI scanner (Achieva, Philips Healthcare, Best, The Netherlands). The projectiles were aligned to the magnetic field lines and perpendicular to the magnetic field lines. In the second experiment, the other four gelatines were placed in a 1.5-T MRI system (Intera, Philips Healthcare, Best, The Netherlands). In gelatine 3, the projectile was placed with the first method to test for possible effects of a simulated trajectory. In gelatine 4, 5 and 6, three different projectiles were place in the two positions in the 1.5 MRI scanner (see Table 3). The effect of the magnetic field on the projectiles was illustrated by subtracting the ‘post-MRI’ MDCT from the ‘pre-MRI’ MDCT image after alignment of both images on the outer contour of the gelatine phantom.

**Table 1:** Description of the used projectiles

Type	Brand	Calibre	Ferromagnetic steel
A	Sellier & Bellot	7.62 × 39 mm	3.59 g
B	Sellier & Bellot	9 mm	Not present
C	Self-made	9 mm	7.6 g

**Table 2:** Results of the first experiment with the 3-T MRI with gelatine number 1 and 2

Gelatine	Projectile	Field strength	Placing projectile	Initial alignment	Detected movement
1	A	3 T	Pushed	Parallel to the *z*-axis	The projectile moved back through the trajectory parallel to the *z*-axis
2	A	3 T	Pushed	Parallel to the *x*-axis	The projectile rotated parallel to the *z*-axis and created a new trajectory parallel to the *z*-axis

**Table 3:** Results of the second experiment with the 1.5-T MRI with gelatine 3–6

Gelatine	Projectile	Field strength	Placing projectile	Initial alignment	Detected movement
3	A	1.5 T	The projectile was suspended on a wire in the liquid gelatine and the wire was subsequently removed	Between the z- and *x*-axis	The projectile rotated parallel to the *z*-axis and made a new trajectory along the *z*-axis
4	A	1.5 T	Pushed	Parallel to the *x*-axis	The projectile rotated parallel to the *z*-axis
5	B	1.5 T	Pushed	Parallel to the *z*-axis	No visible changes
6	C	1.5 T	Pushed	Parallel to the *z*-axis	The projectile moved a few millimetres along the trajectory

## Results

**Fig. 1:**
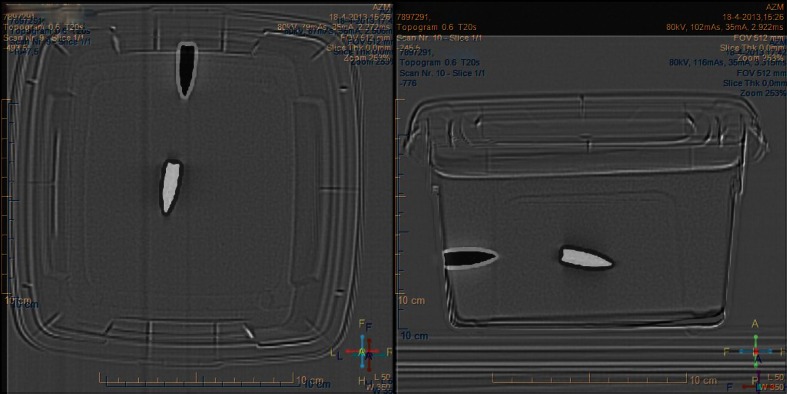
Top view (*left*) and lateral view (*right*) of a MDCT of gelatine 1. The projectile before exposure, the magnetic field of the MRI is shown in *white*; the position of the projectile after exposure to the MRI is shown in *black*. A clear displacement of the projectile has occurred along the direction of the main axis of the static MRI field

**Fig. 2:**
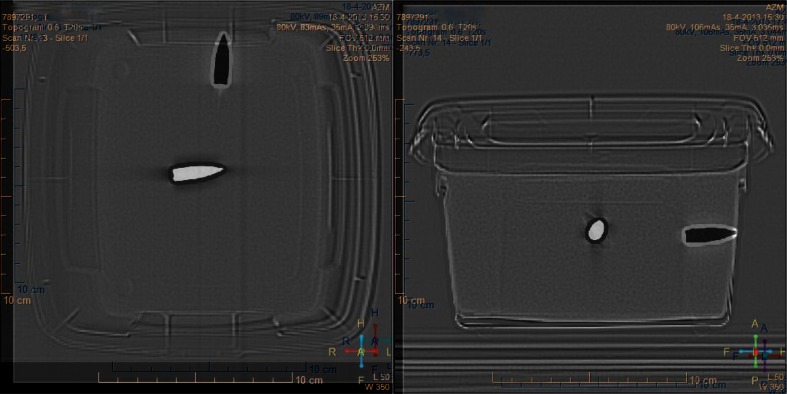
Top view (*left*) and lateral view (*right*) of a CT of gelatine 2. The projectile before exposure, the magnetic field of the MRI is shown in *white*; the position of the projectile after exposure to the MRI is shown in *black*. A clear displacement of the projectile has occurred along the direction of the main axis of the static MRI field in combination with a rotation of the projectile

In the first experiment, the ferromagnetic projectile that was positioned perpendicular to the *z*-axis rotated to a position parallel to the *z*-axis. All projectiles in the gelatine moved along the *z*-axis either along the simulated track or creating a new bullet path (see Table 2 and Figs. 1 and 2). It was observed that most of the movement of the projectiles occurred during the introduction into the bore of the magnet.

Because the extensive movement of the projectiles with 3.59 g of steel in a 3-T magnet, the second experiment was conducted using a 1.5-T magnet.

**Fig. 3:**
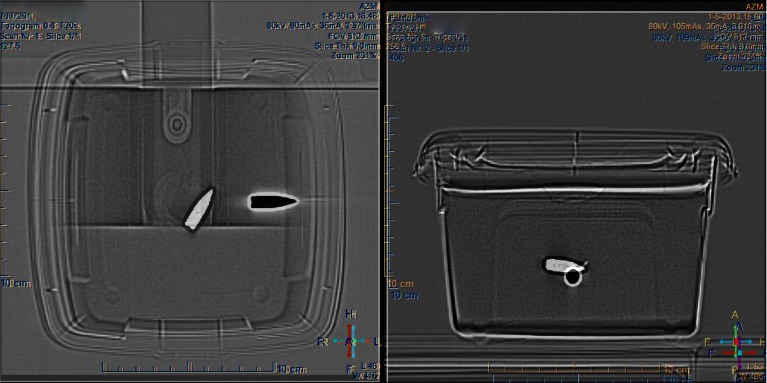
Top view (*left*) and lateral view (*right*) of a CT of gelatine 3. The projectile before exposure, the magnetic field of the MRI is shown in *white*; the position of the projectile after exposure to the MRI is shown in *black*. A clear displacement of the projectile has occurred along the direction of the main axis of the static MRI field in combination with a rotation of the projectile

In the second experiment, the projectile in gelatine 3, without a simulated trajectory, rotated and was displaced in the gelatine (see Fig. 3). In gelatine 4, the projectile aligns with the *z*-axis without movement along the *z*-axis. The non-ferromagnetic projectile in gelatine 5 does not show any movement as expected and the projectile in gelatine 6 shows displacement along the *z*-axis and rotates to a position parallel to the *z*-axis.

**Fig. 4:**
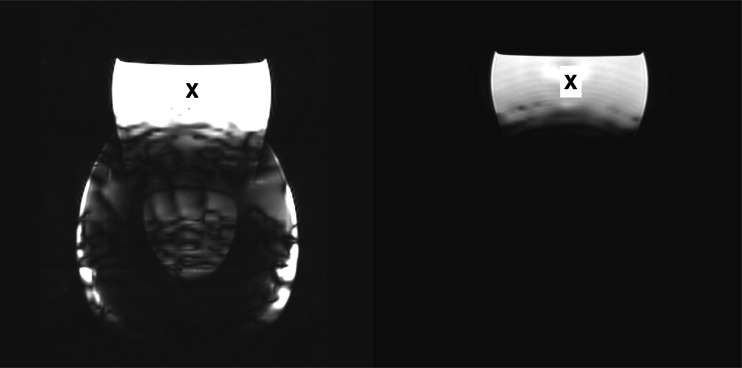
Coronal T1-weighted image (*left*) and T2-weighted image (*right*) of gelatine 1. Note the large susceptibility artefact on both images. The white structure (*X*) is the water phantom on top of the gelatine

The total handling time of gelatine 1 was 100 min and during this time the temperature of the gelatine increased with 3.8 °C. The total handling time for gelatine 3 was 113 min with a temperature rise of 2.3 and gelatine 5 was handled for 110 min with a temperature increase of 2.3. The projectiles in gelatine 1 and 2 gave a large local artefact on T1- and T2-weighted MR images and assessment of the position of the projectile and evaluation of the local anatomy was not possible (see Fig. 4).

## Discussion

The main finding of this study is that both at a magnetic field strength of 1.5 T and 3.0 T ferromagnetic projectiles can rotate and be displaced in a ballistic gelatine phantom. Although the rotation and displacement follow a certain pattern, the movement and rotation is unpredictable. The observation that most movement occurred during the introduction into the bore of the scanner is explained by the fact that the magnetic field gradient is greatest at that point. The forces due to the magnetic field and field gradient on non-ferromagnetic projectiles are typically several orders of magnitude smaller than those on ferromagnetic projectiles, and no movement of non-ferromagnetic projectiles was observed. The observation of the movement of ferromagnetic projectiles has immediate consequences for the application of MRI and makes it unsuitable for forensic radiologic examinations if the presence of ferromagnetic projectiles in the body cannot be excluded. Postmortal displacement of projectiles caused by the static magnetic field of a MRI scanner may affect the accuracy of the assessment of the trajectories, and we have shown that even new trajectories may be formed. This may have consequences for the reconstruction of bullet trajectories and the reconstruction of a crime scene. The discrimination between ante and postmortem trajectories can be based on the presence of a haemorrhage since these will only occur in the living. However, haemorrhage is not the only sign of a trajectory, disruption of the normal anatomy is another important sign. Furthermore the absence of haemorrhage in a specific setting can be a relevant finding because it may be related to a postmortem inflicted injury. The interpretations of focal anatomic disruptions without signs of haemorrhage can be difficult if these anatomic disruptions can be caused by displacement of projectiles due to the magnetic field of a MRI scanner.

Most institutes will perform a postmortem MDCT (PMCT) before a postmortem MRI (PMMR) is considered. A PMCT does not allow for discrimination between ferro- and non-ferromagnetic materials, but PMCT can exclude the presence of metal projectiles.

The risk to living patients with ferromagnetic objects and more specific projectiles and projectile fragments has been described by Eshed et al. who interviewed 17 patients with retained metal fragments of terrorist and combat attacks who underwent an MRI scan [9]. Only one of the 17 patients reported movement of the fragment. The fragment sizes varied from 1 to 10 mm. They also noticed that the difference in size, shape and amount of ferromagnetic metals in part determines the risk. Eshed et al. concludes that conducting 1.5-T MRI examination in patients with retained metal fragments is safe when the fragments are not in the vicinity of vital organs, which has to be confirmed using another imaging technique [9]. Finitis et al. also supports the use of MRI on patients with retained metallic fragments [6]. In 19 patients who underwent an MRI examination, none of them reported any movement of the metal fragment. Teitelbaum et al. reported that MRI poses only a small risk for patients with retained bullets or fragments because the chance that the bullet consists of ferromagnetic metals is very small [5]. Our results show that non-ferromagnetic fragments show no displacement but ferromagnetic projectiles may move within the human body. This depends of course on the local anatomy; ferromagnetic projectiles that are embedded in bone are, for example, less likely to be displaced under the influence of an external magnetic field. It is very likely that retained projectiles and fragments will become more fixed over time due to formation of fibrous tissue surrounding the object. This will also mean that a larger force has to be applied to the object to displace it. Therefore, clinical observations may not necessary be true in a forensic setting, where the projectile is typically only recently introduced. In a recent study, Karacozof et al. assessed the interaction of a 0.30 calibre 7.62 × 39 copper jacketed steel core bullet and a 3-T MRI scanner, the projectile used is similar to the one used in this study [10]. They showed displacement of the projectile, heating and imaging artefacts. Karacozof concluded that this type of projectile is not safe in the MRI [10]. However, they did not use a ballistic gelatine phantom and the mechanical forces were determined with a deflection angle and force measurement.

A limitation of our study is the increased temperature of the gelatine phantom during the imaging procedure. This has potential consequences for the mechanical properties of the ballistic gelatine because an increase in temperature will decrease the force needed to deform and penetrated the ballistic gel. However, the optimal ballistic gelatine phantom simulates muscle tissue and other tissues have different mechanical properties and may have less resistance to deformation and penetrated as compared to muscle tissue. Therefore, the rotation and displacement of the projectile in the gelatine phantom may not have been an optimal simulation of muscle tissue it still shows the possible effects of a projectile in a human body. Another disadvantage is that the use of isotropic materials such as a gelatine phantom does not take into account the heterogeneous nature of human tissues and the different densities of tissues. For example, tendons and fascia sheets will pose a greater resistance to penetrating injury compared to pure muscle tissue, whereas tissues with a lower specific gravity such as long tissue will pose a lesser resistance [11]. Regardless these possible disadvantages, gelatine phantoms have been used to study the ballistic effects of projectiles for many decades (see for a good review [12]). The practical consequence will be that depending on the tissue that surrounds ferromagnetic projectiles in a human body they may or may not move under the influence of an external magnetic field such as a MRI scanner, and movement is not predictable.

## Conclusion

From a forensic point of view, PMMR cannot be used to assess trajectories in gunshot victims if ferromagnetic projectiles or fragments are present in the body. These projectiles may move under the influence of the static magnetic field and even creating new trajectories. This will make a reconstruction of the trajectories in the body and of the reconstruction of the incident as a whole less reliable.

## References

[1] O’Donnell C, Woodford N (2008). Post-mortem radiology—a new sub-speciality?. Clin Radiol.

[2] Thali MJ, Yen K, Vock P, Ozdoba C, Kneubuehl BP, Sonnenschein M, Dirnhofer R (2003). Image-guided virtual autopsy findings of gunshot victims performed with multi-slice computed tomography and magnetic resonance imaging and subsequent correlation between radiology and autopsy findings. Forensic Sci Int.

[3] Tartaglione T, Filograna L, Roiati S, Guglielmi G, Colosimo C, Bonomo L (2012). Importance of 3D-CT imaging in single-bullet cranioencephalic gunshot wounds. Radiol Med.

[4] Hunter TB, Taljanovic MS (2003). Foreign bodies. Radiographics.

[5] Teitelbaum GP, Yee CA, Van Horn DD, Kim HS, Colletti PM (1990). Metallic ballistic fragments: MR imaging safety and artifacts. Radiology.

[6] Finitsis SN, Falcone S, Green BA (1999). MR of the spine in the presence of metallic bullet fragments: is the benefit worth the risk?. AJNR Am J Neuroradiol.

[7] Netherlands Forensic Institute, Handleiding bereiding gelatine voor simulatie menselijk spierweefsel (Guideline for the preparation of gelatines for the simulation of human muscle tissue) QOL-00746 third edition, Den Haag, The Netherlands

[8] Nicholas NC, Welsch JR (2004) Institute for non-lethal defense technologies report: Ballistic Gelatin pp. 1–20

[9] Eshed I, Kushnir T, Shabshin N, Konen E (2010). Is magnetic resonance imaging safe for patients with retained metal fragments from combat and terrorist attacks?. Acta Radiol.

[10] Karacozoff AM, Pekmezci M, Shellock FG (2013). Armor-piercing bullet: 3-T MRI findings and identification by a ferromagnetic detection system. Mil Med.

[11] DeMuth WE (1966). Bullet velocity and design as determinants of wounding capability: an experimental study. J Trauma.

[12] Jussila J (2005) Wound ballistic simulation: assessment of the legitimacy of law enforcement firearms ammunition by means of wound ballistic simulation. Dissertation, University of Helsinki, Finland

